# Pre-clerkship procedural training in venipuncture: a prospective cohort study on skills acquisition and durability

**DOI:** 10.1186/s12909-023-04722-2

**Published:** 2023-10-06

**Authors:** Kaumudee Kodikara, Thilanka Seneviratne, Ranjan Premaratna

**Affiliations:** 1 Department of Medical Education, Faculty of Medicine, University of Kelaniya, Ragama, Sri Lanka; 2 Department of Pharmacology, Faculty of Medicine, University of Peradeniya, Peradeniya, Sri Lanka; 3 Department of Medicine, Faculty of Medicine, University of Kelaniya, Ragama, Sri Lanka

**Keywords:** Procedural training, Simulation-based training, Medical student, Communication skills

## Abstract

**Background:**

The effectiveness of simulation-based training for skill acquisition is widely recognized. However, the impact of simulation-based procedural training (SBPT) on pre-clerkship medical students and the retention of procedural skills learned through this modality are rarely investigated.

**Methods:**

A prospective cohort study was conducted among pre-clerkship medical students. Learners underwent SBPT in venipuncture in the skills laboratory. Assessments were conducted at two main points: 1) immediate assessment following the training and 2) delayed assessment one year after training. Learner self-assessments, independent assessor assessments for procedural competency, and communication skills assessments were conducted in both instances. The students were assessed for their competency in performing venipuncture by an independent assessor immediately following the training in the simulated setting and one-year post-training in the clinical setting, using the Integrated Procedural Protocol Instrument (IPPI). The student’s communication skills were assessed by standardized patients (SP) and actual patients in the simulated and clinical settings, respectively, using the Communication Assessment Tool (CAT).

**Results:**

Fifty-five pre-clerkship medical students were recruited for the study. A significant increase was observed in self-confidence [mean: 2.89 SD (Standard Deviation) (0.69)] and self-perceived competency [mean: 2.42 SD (0.57)] in performing venipuncture, which further improved at the delayed assessment conducted in the clinical setting (*p* < 0.001). Similarly, the IPPI ratings showed an improvement [immediate assessment: mean: 2.25 SD (1.62); delayed assessment: mean: 2.78 SD (0.53); *p* < 0.01] in venipuncture skills when assessed by an independent assessor blinded to the study design. A significant difference (*p* < 0.01) was also observed in doctor-patient communication when evaluated by SPs [mean: 2.49 SD (0.57)] and patients [mean: 3.76 SD (0.74)].

**Conclusion:**

Simulation-based venipuncture training enabled students to perform the procedure with confidence and technical accuracy. Improved rating scores received at a one-year interval denote the impact of clinical training on skills acquisition. The durability of skills learned via SBPT needs to be further investigated.

**Supplementary Information:**

The online version contains supplementary material available at 10.1186/s12909-023-04722-2.

## Background

The achievement of clinical competency is a gradual process, with repetitive training being a central element in the continuum of medical education [1, 2]. The pre-clinical period fraught with teaching basic sciences, is used less to equip students with skills needed at the bedside to participate in patient care during clerkships [3]. Thus, clerkships are still the primary source for learning and acquiring clinical skills in traditional medical curricula [4–6]. However, traditional curricula are no longer recommended, and many medical schools have undertaken curricula reforms to move towards integrated curricula [7].

However, basic clinical skills acquisition during clerkships occurs in a rather "haphazard" fashion [6, 8–12]. Practicing invasive procedures on patients without proper training imposes an ethical issue [13]. A growing number of learners, finite resources, and increasing emphasis on patients' right to trained care hinder medical students' learning procedural skills in the clinical setting [14]. Further, students report inadequate supervision by the clinical teachers, lack of assessments and feedback on learner performance, and reduced opportunities for learning [6, 8, 12] as barriers to learning procedural skills at the bedside. Although patients are willing to accept trainee involvement in nonprocedural care, they usually are reluctant to allow medical students to perform procedures on them [15, 16]. Therefore, the opportunity to develop basic procedural skills in the ward-based setting has become a challenge.

Consequently, several studies report a lack of clinical experience and competency in performing essential procedures by medical students and resident physicians [17–21]. In a single-center study, residents experienced a discrepancy between the actual and desired competency levels for basic procedural skills [22]. However, mastering these procedures is essential for medical students [23–25]. Hence, to bridge the gap between expectations and learning experiences in clinical clerkships, simulation-based procedural training (SBPT) has been increasingly integrated into medical curricula [26, 27].

Hence, SBPT in skills laboratories has taken on a central role in training procedural skills. SBPT allows students to learn in a safe environment where they can engage in deliberate practice to achieve proficiency [28]. Teaching/ learning with SBPT is usually structured and employs different instructional approaches, including the "Four-Step Approach" devised by Rodney Peyton [29, 30]. Each learning session is reinforced by a debrief session, where students are encouraged to reflect upon their performance fortified by educational feedback, a unique feature of simulation-based medical education [31]. SBPT employs part-task trainers [32], peers or near peers [33], and hybrid simulators (part-task trainers coupled with Standardized Patients- SPs) [34].

The effectiveness of SBPT is widely recognized. Compared with standard or no training, SBPT was found to enhance learner competency [35] and improve the performance of basic clinical skills when assessed in OSCEs [36, 37]. Peer-led learning has demonstrated effectiveness in skills acquisition equal to teacher-led instruction with SBPT [38, 39]. SBPT has led to an increase in the number of procedures students perform in the wards [40]. Thus, Remmen et al. assumed that skills training better prepares students for clinical clerkships [41]. Students were found to be less anxious and more confident at the bedside with procedural training in the pre-clerkship period [42]. Therefore, SBPT is recommended to be integrated as a longitudinal training course into medical curricula [43], starting from the pre-clerkship period [44].

In contrast to a growing literature on procedural performance among undergraduates in the West [45, 46], there has been no previous objective skills assessment of undergraduates in South Asia, where the curricula, resources, and educational opportunities are in stark contrast. Specifically, we did not find evidence of implementation or the effectiveness of a pre-clerkship SBPT course available for medical students across the South Asian subcontinent, including Sri Lanka. In addition, literature on the retention of procedural skills acquired through SBPT lacks robust evidence [35, 46–50], with critical reviews of simulation for procedural skills training rarely conducted in the last decade [45, 51]. Despite the well-established phenomenon of technical skill decay [52], no study has assessed procedural skill retention in this population. The limited available research on the natural history of technical skills among undergraduates has focused on basic and advanced life support [53, 54], with recommendations to investigate the skill decay in relation to context and tasks [46].

We aimed to address two gaps in the literature. This study aimed to assess the impact of a simulation-based procedural skills training program among pre-clerkship medical students. Second, we aimed to measure the durability of medical students' venipuncture skills. Specifically, the study asks the following questions: 1) Do pre-clerkship medical students demonstrate improved self-confidence and perceived competency with simulation-based venipuncture training? 2) Do pre-clerkship medical students demonstrate competency in technical and communication skills in performing venipuncture when assessed by an independent assessor? 3) Do students retain the skills learned through SBPT when assessed by an independent assessor at a one-year interval?

## Methods

### Context of the study

Sri Lanka, the setting for this study, is a South Asian island nation with its' medical education influenced by the British [55]. All medical schools in Sri Lanka are affiliated with public Universities. Eleven government-funded Universities that provide undergraduate medical education, including the Faculty of Medicine, University of Kelaniya, where this study was conducted, have undergone curricula reforms to shift away from traditional didactic methods, advocating for student-centered teaching–learning approaches [55]. However, most of these changes focus on delivering the taught curriculum, with minimal attention to teaching/learning methods used during clinical training.

The undergraduate medical curricula of Universities in Sri Lanka, including where we conducted this study, comprise five years. The medical course is divided into a 2-year pre-clinical, 2-year para-clinical, and 1-year clinical phases. The pre-clinical phase included no clinical contact and was focused on teaching basic sciences. At the time of the study, most medical schools, including the University of Kelaniya, were equipped with skills laboratories where simulation-based procedural and communication skills training were conducted to varying extents. A single skills laboratory group at the University of Kelaniya would have about 60 students. Opportunities for redundant training and deliberate practice are virtually nonexistent due to the resource-limited nature of the local context. The few procedures trained during the pre-clinical phase are thus not revisited in the following years. Although these skills laboratory classes were mandatory, the skills taught were not formally assessed.

The focus of the para-clinical phase was on teaching applied sciences. In affiliated state hospitals, these students participated in half-day clinical rotations in General Medicine, General Surgery, Pediatrics, Gynecology and Obstetrics, Psychiatry, and related subspecialties. The educators rely on the clerkships for students to learn and practice procedural skills, which start after a 21-month (mean) interval.

The clinical phase was entirely dedicated to clinical rotations in General Medicine, General Surgery, Pediatrics, Gynecology and Obstetrics, and Psychiatry. Each clinical rotation between years 3–5 is 4–8 weeks, with students 'attached' to one or more consultants in the ward/unit. During the clerkships, the students are required to achieve procedural competency by observation, legitimate peripheral participation [56], and practicing procedures on actual patients. A single clerkship group (years 3–5) at the institution where we conducted this study consisted of 30–40 students.

### Study setting

A prospective cohort study [57] was conducted among pre-clerkship medical students in a metropolitan University in Sri Lanka from 2020–2021. The study focused on venipuncture, a basic procedural skill required of a resident physician. All second-year medical students who agreed to participate were included in the study.

The study was conducted in two phases. In phase I, all 55 second-year medical students of the Faculty of Medicine, University of Kelaniya, Sri Lanka, who volunteered and were eligible for the study, were recruited. All students underwent SBPT on venipuncture using hybrid simulators (part-task trainers coupled with SPs) in the skills laboratory. The self-confidence and perceived self-competency in performing venipuncture were assessed before and after the training. An independent assessor assessed venipuncture performance, and the SPs assessed communication skills.

**Fig. 1:**
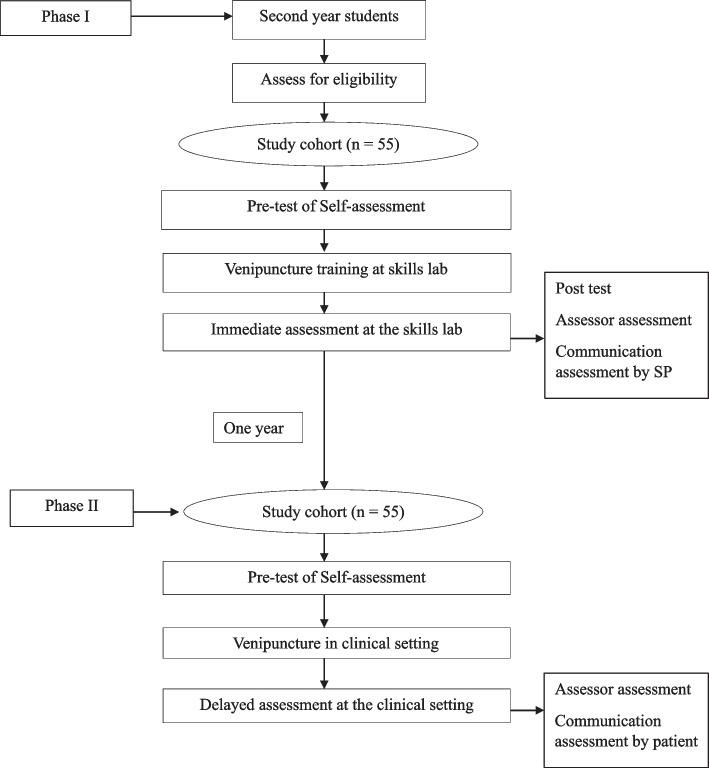
Study flow chart

In phase II of the study, this cohort of students was re-assessed in the clinical setting one year after SBPT. The students rated their self-confidence and perceived self-competency. Subsequently, they performed venipuncture on actual patients, and an independent assessor assessed the skills. Actual patients assessed their communication skills in the clinical setting. Figure 1 demonstrates the methodology.

### Study participants

#### Student sample

The primary inclusion criterion was second-year medical students at the Faculty of Medicine, University of Kelaniya. Students with previous experience performing procedures such as venipuncture, IV cannulation, or intravenous injections were excluded from the study. Information about these exclusion criteria and student characteristics (age and gender) were obtained through a self-administered questionnaire handed over to the students one week before the commencement of the study. We were intentionally inclusive to give all volunteering second-year medical students the opportunity to receive training.

#### Standardized patients (SP) sample

SPs were recruited for phase I of the study. They were coupled with mannequins to enable role-play. All SPs who acted as patients in the study received written role-play instruction. The SPs were used in the simulated setting for the participants to learn communication skills concerning venipuncture while performing venipuncture on the task trainer. SPs were instructed on using the assessment tool to evaluate the student's communication skills.

#### Patient sample

Patients were recruited for the study in phase II. Individuals taking anticoagulant drugs, diagnosed patients with Hepatitis B, C, or HIV, critically ill patients, and patients unable to give written consent were excluded from participation. In addition, patients diagnosed with coagulopathies and heavy smokers were also excluded from the study. Only patients indicated for blood sampling were recruited for the study. These details were gathered from the bed-head tickets of patients, and only the eligible patients were requested to participate in the study.

### Ethics

Ethics approval was granted by the ethics review committee of the Faculty of Medicine, University of Kelaniya (P/233/11/2019). Informed written consent was obtained from all volunteering students, SPs, and patients. Further, permission for conducting the study in the clinical setting was obtained from the Director of the Colombo North Teaching Hospital and relevant Consultants in the wards. Study participation was voluntary, and all participants were assured of anonymity and confidentiality. The student participants were ensured that the participation, withdrawal from the study, or assessment scores they received during the study would not affect them in any way in their clinical training and assessments. All volunteering participants were allowed to refuse or withdraw from the study at any time. They were assured that refusal to participate or withdrawal from the study would not affect the care and treatment they received from the ward. All criteria were applied in order to minimize risks of potential harm for both students and volunteer patients.

### Phase I

#### Pre-interventional questionnaire for student participants

Students were given a self-administered questionnaire to gather baseline data (i.e., age, gender) and to rate their self-perceived confidence and competency levels in performing venipuncture on actual patients. The questionnaire was developed from published literature [37, 58]. We pre-tested the developed questionnaire with a selected group of ten first-year medical students and five clinicians who were medical educators. Although second-year medical students would best represent the study participants, we could not invite them due to the possibility of any one of them being included in the study. We chose clinician academics for the pre-test group to further improve the quality of the questionnaire. The pre-test group participants were asked to complete and critique the questionnaire using several criteria, including the adequacy of instructions, clarity of questions to identify incongruent and vague statements, comprehensiveness, and rating methods. Pre-testing was done to ensure the relevance and acceptability of the participating students. In addition, the pre-test group was asked to suggest corrections and recommendations for inclusion in the instrument. The modified and refined questionnaire was used for data collection.

The students were asked to rate their self-confidence on a 5-point Likert scale (1 = beginner, 5 = master). The students rated the overall self-perceived competency to perform venipuncture on a 4-point Likert scale which ranges from 1-unable to perform, 2 = competent to perform with major assistance, 3 = competent to perform with minor assistance, to 4 = competent to perform independently. *Major assistance* was defined as assistance required in one or more of the three major steps considered essential in performing the task; a) selecting the vein, b) selecting the site of venipuncture, or c) insertion of the needle into the vein. *Minor assistance* was defined as needing assistance in one or more of a) asepsis, b) tying the tourniquet, c) dressing the venipuncture site, etc.

#### Intervention

The cohort of second-year medical students recruited to the study underwent SBPT in venipuncture in the skills laboratory during 2-h training sessions. The training was conducted in small groups. Each training group consisted of 3–4 students per hybrid simulator. They also revisited and trained on venipuncture-associated patient-physician communication during the training session. The intervention was carried out as a role-play using a hybrid simulator. The part-task trainer was used to train the technical skills of venipuncture. The SP was used for learners to practice communication during venipuncture.

The students received instruction on the technical aspects of venipuncture according to Rodney Peyton's 'Four-Step Approach' [30]. They trained on venipuncture on a part-task-trainer model in the shape of a human arm (serial number: 312029 T; name: Multi-Venous IV Training Arm", purchased via Laerdal, New York, USA).

The students were trained in communication skills using SPs with whom they practiced doctor-patient communication. They had been trained in communication skills using the Agenda Led Outcome-Based Analysis scheme (ALOBA) developed for simulation-based learning of communication skills [59] prior to the study's recruitment. During the intervention, the instructors revisited the concepts of doctor-patient communication. They encouraged learners to practice venipuncture-associated communication skills with the SP while performing venipuncture on the task trainer. The exercise was carried out as a role-play to create a more realistic environment that enhanced the student's involvement in the learning experience and to support the acquisition of doctor-physician communication [33]. The SPs were given detailed role-play instructions by the instructor.

After the instructor demonstrated the procedure using the hybrid simulator, the instructor allowed the participant to practice on the simulator while providing direct, specific feedback on technical performance and communication. We allowed the participants to practice as many times as they desired, either on particular steps or on the entire procedure from start to finish. Per mastery learning practices [60], they iteratively received direct feedback and targeted practice on the steps that were not achieved until they could independently complete the entire procedure. Equal emphasis was placed on the self-contained, practical exercise of venipuncture on a part-task trainer and doctor-patient communication.

#### Outcome assessments

The participants were assessed for their competency in performing venipuncture in two instances: immediately following the training session and as a delayed assessment. The immediate assessment took place following the conclusion of the venipuncture training sessions. The participants performed venipuncture using hybrid simulators at the clinical skills laboratory. The delayed assessment occurred at the clinical (i.e., ward) setting one-year post-venipuncture training, where the participants were requested to perform venipuncture on actual patients. The outcomes measured in both instances were: 1) self-assessments of confidence and competency, 2) independent assessor assessment of procedural competency, and 3) SP/patient assessment of the communication skills. Additionally, the number of procedures each student recalled performing in the prior year was collected using the self-administered questionnaire in phase II of the study that gathered data on self-confidence and self-perceived competency.

1. Assessment of self-confidence and self-competency
  Post-intervention, the same pre-test questionnaire was given again. Participating students rated their confidence and competency in performing venipuncture on real patients. The students rated their self-confidence on a 5-point Likert scale (1 = beginner, 5 = master) and the overall self-assessed competency to perform venipuncture on a 4-point Likert scale (1-unable to perform, 2 = competent to perform with major assistance, 3 = competent to perform with minor assistance, 4 = competent to perform independently). In addition, they were also asked about their perception of the teaching session in an open-ended question.
2. Assessment of trained skills by an independent assessor
  Students' performance was assessed by an independent assessor blinded to the study design. Using an independent and blinded assessor removes possible bias in assessment. The assessor was an experienced clinician actively involved in medical student education with vast experience in teaching and assessing clinical skills. The assessor received instruction on how to use the IPPI from the principal investigator of this project prior to the commencement of the study.
  This assessment was conducted using the Integrated Procedural Performance Instrument (IPPI) proposed by Kneebone et al. [61]. The IPPI is designed to assess procedural competencies where task trainers are combined with SPs to better approximate actual clinical situations. IPPI consists of nine items: introduction/establishing rapport, explanation of the procedure, consent, preparation for the procedure, technical performance of the procedure, maintenance of asepsis, closure of procedure, professionalism, and overall ability to perform the procedure (technical and professional skills). The performance is graded as below average to above average (Additional file 1). In this study, we divided the IPPI into three main subcategories as items that describe "technical aspects," items that mainly describe "communication skills," and items that describe the overall ability as "overall performance," and a sub-analysis was conducted.
  All participants were given a maximum of three attempts to perform venipuncture, after which the student was refrained by the facilitator from performing the procedure.
3. Assessment of communication skills by SPs
  The SPs assessed the students' performance with a modified and translated Communication Assessment Tool (CAT). CAT, developed for patients to rate clinicians' interpersonal and communication skills, has shown evidence of validity in various contexts [62]. The translated and modified CAT was pre-tested and piloted prior to the commencement of the study. SPs were trained by the principal investigator to assess communication skills using the translated CAT.

### Phase II

#### Delayed assessment of competency in the clinical setting

The cohort of pre-clerkship students who received SBPT on venipuncture was recruited back to the study one-year after venipuncture training. This one-year was mainly dedicated to pre-clerkship learning and end-of-year assessments. By the time they were recruited to the study, they had proceeded to the 3rd year of the medical course. They had undergone one month of clerkships in medical or surgical wards at the Colombo North Teaching Hospital, Sri Lanka. At the time of this assessment, the participants have performed venipunctures on real patients, with a frequency ranging between 2–5 venipunctures per student during the month of training in their medical or surgical rotation.

1. Assessment of self-confidence and self-competency

Before performing venipuncture on real patients, the students were given the same questionnaire used to assess the level of self-confidence and perceived self-competency in performing venipuncture on an actual patient.

2) Assessment of trained skills by an independent assessor

All study participants performed venipuncture on real patients in the clinical setting. The participants were given only a maximum of two attempts for venipuncture under the supervision of the principal investigator or a qualified, trained medical officer.

The same independent assessor who was blinded to the study design assessed the students' performance. This assessment was conducted using the IPPI [61].

3) Assessment of communication skills by real patients

The patients rated the students' communication skills using the CAT [62]. The questionnaire was interviewer-administered. Extended faculty staff members blinded to the study design were responsible for administering the questionnaire to the patients.

### Statistical analysis

In this study, medical students served as their own controls for statistical analyses [57]. Comparisons between pre and post-intervention variables were made using Wilcoxon signed rank test. Data are presented as means with standard deviations (SD) and medians with 25th and 75th centiles. Distribution of group characteristics referring to age and gender are presented as percentages. Responses for each variable were tested for normality by the Shapiro-Wilks test. The Wilcoxon sign rank test was used to compare the means of pre-and post-interventional variables, including self-assessments and IPPI, and the medians of CAT ratings. G*Power was used to estimate that a sample of 26 would be sufficient to detect an effect size of Cohen's d value of 0.45 (α = 0.05) with 80% power for Wilcoxon signed rank test. A P-value less than 0.05 was considered to be statistically significant. Raw data were processed using Microsoft EXCEL. SPSS software version 22 (Armonk, NY) was used for statistical analysis.

## Results

### Group characteristics of the study participants

All 55 students recruited to phase I of the study were second-year medical students. The student group comprised 35 (63.64%) female and 20 male participants. The mean age was 21.78 ± 0.98 years.

### Students’ self-assessment ratings

#### Pre-intervention

Most participants (*n* = 45; 81.82%) rated their self-confidence to perform venipuncture as beginner. None of the students rated their self-confidence at the level of ‘Master.’ Most students (*n* = 39; 70.91%) felt they knew the steps but could not describe the steps in performing venipuncture.

Most students (*n* = 31; 56.36%) felt they could not perform the skills independently. Whereas 41.82% felt they could perform venipuncture with major assistance.

#### Post-intervention

The majority of the students (*n* = 29; 52.73%) felt their self-confidence increased to level 3 from level 1 (beginner) following SBPT. Although none felt they reached the level of master following the training, ten students (18.18%) felt their self-confidence increased to level four. 40% felt they could perform with minor help, while 56.36% stated their self-competency as being able to perform venipuncture under supervision with major assistance. A statically significant mean increase in self-confidence and self-competency was observed in the participants following SBPT (*p*-value < 0.05).

#### Self-assessment in the clinical setting

When assessed for self-confidence to perform venipuncture, one year after venipuncture training in the skills lab, most (*n* = 38; 69.10%) of the participants rated self-confidence as level four. In addition, 20% rated their self-confidence to have reached the level of master in performing venipuncture. Most students (*n* = 44; 80%) felt they could perform the skills independently. Table 1 compares the mean scores of self-ratings.

**Table 1:** Comparison of self-ratings

Self-assessment	Simulated setting	Clinical setting	*p-*value
	**Mean (SD)**	**Mean (SD)**	
Self-confidence	2.89 (0.69)	4.09 (0.55)	< 0.001
Self-competency	2.42 (0.57)	3.80 (0.40)	< 0.001

### IPPI ratings

#### Post-intervention (simulated setting)

The assessor rated the overall performance of the students as competent/ borderline or incompetent. Most participants were rated borderline (*n* = 28; 50.91%), and 41.82% were rated competent. Interestingly, four students (7.27%) were rated incompetent to perform venipuncture following simulation-based training. These students refrained from performing venipuncture after three attempts. They were given remedial training after the conclusion of the training and assessment of the rest of the group. Amongst these four students, three were rated borderline, and one was rated competent when assessed by the same independent assessor after the completion of the remedial training session.

#### Clinical setting

When assessed one year later, the overall performance in venipuncture of the participants was rated as competent (*n* = 40; 72.7%). Table 2 compares the IPPI ratings given to participants in the simulated setting following SBPT and the delayed assessment conducted in the clinical setting.

**Table 2:** IPPI ratings of the participants in performing venipuncture. Data are presented as numbers and (percentages)

IPPI sub-component		Below expectations	Borderline	Meets expectations	Above expectations
***Communication aspects***					
Rapport	SS^a^	2 (3.64)	28 (50.91)	22 (40)	3 (5.45)
	CS^a^	0	7 (12.7)	46 (83.6)	2 (3.6)
Explaining the procedure	SS	0	33 (60)	19 (34.55)	3 (5.45)
	CS	0	2 (3.3)	52 (94.5)	1 (1.82)
Taking consent	SS	0	35 (63.64)	19 (34.55)	1 (1.82)
	CS	0	4 (7.3)	50 (90.9)	1 (1.82)
Closure	SS	0	32 (58.18)	21 (38.18)	2 (3.64)
	CS	0	5 (9.1)	50 (90.9)	0
***Technical aspects***					
Preparation for the procedure	SS	0	26 (47.27)	27 (49.09)	2 (3.64)
	CS	0	7 (12.7)	48 (87.3)	0
Technical performance of the procedure	SS	0	25 (45.45)	26 (47.27)	4 (7.27)
	CS	0	17 (30.9)	38 (69.1)	0
Maintaining asepsis	SS	1 (1.82)	30 (54.55)	21 (38.18)	3 (5.45)
	CS	0	3 (5.5)	52 (94.5)	0
***Overall performance***					
Professionalism	SS	0	34 (61.82)	19 (34.55)	2 (3.64)
	CS	0	3 (5.5)	51 (92.7)	1 (1.8)
Overall ability	SS	0	27 (49.09)	24 (43.64)	4 (7.27)
	CS	0	6 (10.9)	48 (87.3)	1 (1.82)

^a^*SS* Simulated setting, *CS* Clinical Setting

A significant difference was observed between the two settings across all categories and subcategories of the IPPI, as shown in Table 3.

**Table 3:** Comparison of IPPI ratings

IPPI subcategory	Simulated setting	Clinical setting	*p-*value
	**Mean (SD)**	**Mean (SD)**	
Communication aspects	10.24 (2.03)	11.75 (0.87)	< 0.01
Technical aspects	7.35 (1.52)	8.51 (0.74)	< 0.01
Overall ability	5.00 (1.09)	5.87 (0.58)	< 0.01
Overall performance	2.25 (1.62)	2.78 (0.53)	< 0.01

### CAT ratings

Rated on a scale of 1–5 (poor-excellent) by SPs on doctor-patient communication, the median score for study participants was 3.0, corresponding with the "fair" response. When rated by patients in the clinical setting, the students were rated as good (median: 4.0; *p* < 0.01).

### Medical students’ reaction to simulation-based training

Most students (*n* = 50) indicated they were satisfied with the learning experience. Students felt that this learning environment motivated learning and that they felt prepared for the clerkships owing to this experience. Most students recommended this training to the rest of the medical students in the pre-clinical phase. They suggested that the training be conducted for other common procedures they would encounter in the clinical setting.

## Discussion

Our work is the first to document the impact of SBPT on procedural competency among pre-clerkship medical undergraduates in South Asia. Our cohort study of pre-clerkship medical students undergoing SBPT for venipuncture demonstrated significant improvements in self-assessment and procedural competency. They reported enthusiastic and positive attitudes toward SBPT. Although we expected decreased scores for competency and self-assessments in the delayed assessment, we noted improved ratings when assessed for competency one-year after training.

We were not surprised by the baseline (pre-intervention) self-ratings of confidence and competency of the students before SBPT since these students were in the pre-clerkship period and, therefore, were not exposed to procedural training. Important to note are the ratings of the post-SBPT IPPI. In a BEME systematic review, Issenberg et al*.* (2005) showed simulator validity and feedback as critical features of simulation-based training, which leads to “most effective learning” [1, 27, 31]. In our study, the validity of the SBPT was improved by incorporating SPs which led to a learning exercise through role play. Role-playing enhances the realism of skills training and aids in learning doctor-patient communication during the training sessions [33]. In addition to incorporating role play, we provided immediate constructive feedback to the students. Both these features may also have contributed to the observed results in this study.

Previous work in evaluating the effectiveness of SBPT identified that the inclusion of several procedures within a single study limited the time and capacity for proper assessment [37]. In addition, incorporating video assessors also have inherent difficulties with logistical challenges to capture each student’s performance in a high-quality video that provides a detailed view of all necessary angles for an accurate procedural skills assessment [37, 63]. Thus, this study was planned to mitigate these issues by using real-time skills assessment of a single procedure, which enabled us to gather robust data in this study.

Although the effects of pre-clerkship SBPT are well established in the West [45, 46], evidence for simulation-based education among pre-clinical medical undergraduates in Asia, where the curricula, resources, and educational opportunities are at a stark contrast, are lacking. Of note, a study from East Asia showed a marked improvement in procedural competency with SBPT for clerkship students [64].

Our study demonstrated and confirmed satisfactory technical and communication skills gain among pre-clerkship medical students. The students in this study were enthusiastic and positive toward SBPT, which reflects the existing literature on learner satisfaction with simulation [45]. Pre-clerkship procedural training in Sri Lanka remains ad-hoc, and currently, work is underway to identify essential procedural skills competencies required as exit qualifications from the undergraduate medical program. The findings in this study contribute to this endeavor to develop a pre-clerkship procedural training course for undergraduate medical curricula.

The second aspect we wanted to investigate was the durability of skills gained through simulation-based training. Opportunities for re-training and deliberate practice are virtually nonexistent for medical undergraduates in Sri Lanka due to the resource-limited nature in the local context. The few procedures trained during the pre-clinical phase are thus not revisited in the following years. The educators rely on the clerkships for students to learn and practice procedural skills, which start after an 21-month (mean) interval. Therefore, we expected to investigate whether students would benefit from skills training way before the start of clerkships and how much of a skills retention we could observe one year after training, a phenomenon investigated in postgraduate medical education [65, 66]. The findings of this study have the potential to inform current educational practices and instructional design with high implications for the local context, which also applies to similar settings.

Consequently, we were highly surprised by the improved self-assessment and IPPI ratings reported during the delayed assessment. We expected medical students to be unable to sustain procedural competency when assessed a year later due to a lack of ongoing experience. After a one-year gap, we also expected diminished self-confidence and perceived competency levels to perform skills. The ratings on communication skills by the SPs differed significantly from those of the patients, a finding we anticipated in the study. This finding complies with literature where patients are reported to rate the students’ performance more benevolently than SPs [67].

Many studies have investigated skill retention in relation to cardio-pulmonary resuscitation skills or advanced cardiac life support skills following simulation-based training [54, 68]. Studies on simulation-based training of postgraduate doctors on hemodialysis [65] and lumbar puncture [66] have shown to retain skills one-year after training. Notably, in undergraduate medical education, Lee and colleagues recruited ten medical students who had undergone a single simulation-based training on cardiovascular system examination one year ago [69]. These students have not had further training after the initial training session. The cardiovascular examination skills of the students were evaluated through MCQ (Multiple Choice Questions) and OSCE (Objective Structured Clinical Examination) one year after training. They concluded that the students were able to retain the skills learned through simulation-based training for one year despite the lack of training in between. However, more recent studies have shown evidence of steep skills decay following SBPT [70], with recommendations for booster training at intervals to maintain procedural competency [65, 71].

Practicing invasive procedures without proper training imposes an ethical issue [13]. It was deemed unethical to request students to perform venipuncture on actual patients with training limited to SBPT on venipuncture one year ago. Thus, we expected a skill decay in accordance with previous research [70, 71]. Hence, to overcome the ethical issues arising from requesting students (who only had SBPT on venipuncture one year ago and thus, deemed not to have adequate exposure) to perform venipuncture on actual patients, we designed the study so that the students were given four weeks of clinical training where they would be able to perform venipuncture on actual patients.

The improved ratings reported in the delayed assessment were highly intriguing. This effect goes against the principle of deliberate practice by McGaghie [28]. We speculate that the improved ratings received in the delayed assessment cannot be directly related to the effects of SBPT. Due to the design of this study, the participants’ skill decay may have been masked by superimposed clinical training, albeit one month, and low procedural volumes. The number of venipunctures our participants self-reported in the clerkship month prior to the second assessment was quite low (2–5 venipunctures per student), which, if representative of the procedures available to them, they would have had a scarce opportunity to benefit from the booster effect of procedural volume on skill refinement [72].

The improved ratings we observed in the delayed assessment are unlikely to be an effect of the SBPT they received a year back. Although the practice opportunities were low, it is possible that they were motivated to learn and perform better after being recruited to the study in the second phase. Knowing they might have to perform for the study may have improved efforts to learn. However, we foresaw the possibility of the Hawthorn effect [73] and minimized it by reducing the time between recruitment and assessment to a maximum of five days. We could also argue that the students were accustomed to the local context, where they had to learn and perform procedures with minimal training, which may allow us to generalize this surprising finding to the larger student body. Another possibility is the effect the raging COVID-19 pandemic had on medical students’ learning. We conducted this study at the height of the pandemic when non-COVID admissions were low and clinical teachers were heavily burdened by the increased workload, taxing the typical ‘ward classes’. Thus, students had more time than was standard during these clerkships to be involved with more hands-on learning, including procedural practice, which may have been reflected in the results of the second phase of this study. We, as researchers, wish to disseminate this unusual finding in the hope that these results may open avenues to discuss current educational practices and what works for different learner communities. Nevertheless, we are cognizant of the many confounding factors that hindered a robust evaluation of procedural skills retention, and a randomized controlled trial is on the way to evaluate the same.

Our study findings also comply with the concept of situated learning theory [74]. In the SBPT on venipuncture, the students could just insert the needle without manipulating the mannequin’s skin. In the clinical setting, they encountered soft skin and veins that looked and were positioned differently. These differences in the conditions and appearances required the students to assess the patient’s vein by touching, checking, and choosing the most appropriate vein. Although this is different from the learning at the skills laboratory, the students were able to grasp new experiences and construct new knowledge [74].

We used the IPPI to evaluate students’ performance in simulated and clinical settings. IPPI has been developed by Kneebone et al. (2006) based on DOPS (Direct Observation of Procedural Skills) for use in a simulated setting for teaching and assessment of clinical procedures where technical skill and professional behavior are given equal value. Although tools such as DOPS and Observed Structured Assessment of Technical Skills (OSATS) have been validated to be used in the clinical setting for procedural assessment [75, 76], the use of such tools for procedural skills assessments at the undergraduate level is limited [77]. Moreover, DOPS and IPPI have been used interchangeably in both the simulated and clinical settings [63, 76, 78]. Some salient features in IPPI were on par with our study component in the clinical setting (e.g., patient providing feedback, no engagement of the assessor and the student, and assessor unknown to the student). Furthermore, given the pre-post design of our study, we opted to use the IPPI in both settings to facilitate comparisons and draw on conclusions. Additionally, Kneebone recommended comparing IPPI with DOPS outcomes [61]. We aim to investigate the alignment between IPPI and workplace-based DOPS in a cross-over study to extend the valuable work by Kneebone. This would advance our understanding of the relationship between clinical procedures in real and simulated settings.

Our study shed light on the impact of pre-clerkship procedural training through simulation. However, it opened room for deliberation about what, why, and how procedural training worked for this largely overlooked study population. We also highlight the practical realities that must be overcome to extend this work to generate robust evidence on the retention of skills acquired through SBPT.

### Limitations

Several limitations of our study should be mentioned. This study was carried out in a single institutional setting with a single cohort of students. Although the students themselves were taken as the controls, a case–control design may produce further insight into the effectiveness of procedural skill acquisition through this learning modality.

All 55 second-year medical students who took part in the study were volunteers. Thus, they might have had bias or interest toward simulation-based training compared to the larger population of students. Hence, students’ positive attitudes toward SBPT reported in this study may not apply to the larger student population.

Our study assessed the effectiveness of venipuncture skills training among pre-clerkship medical students. The results of this study could be applied to the larger domain of procedural skills, especially in relation to techniques that require venipuncture (i.e., cannulation, blood cultures). Thus, the generalizability of our results to less related techniques needs to be evaluated by future studies.

Considering that the students have undergone a month of clerkships, the findings of the delayed test do not say anything in terms of the effect of the intervention. The findings in the delayed tests may not be an accurate proxy of skills retention as many confounding factors, such as prior clinical training, appear to have masked the expected skills decay. However, we reported these unique and early-stage findings in an overlooked line of inquiry to inform future research. Although unexpected, the improved ratings in the delayed assessment require further investigation in terms of understanding what factors were at play to generate the findings of this study.

Although the recommendation is to use multiple assessors [79], we had to rely on a single assessor due to the lack of human resources for the study. However, we used the same assessor for both assessments to minimize the bias. We did not control the procedural volume during the clerkship month between training and retesting, which disclosed the extent to which students had occasion to apply their training, which may have affected the ratings received in this study.

Other than the age and gender, we did not collect data on the number of times needed to accurately perform the procedures, time to completion or complication rates, or past examination performance to extrapolate a possible generalizability of the results.

## Conclusions

The SBPT on venipuncture allowed medical students to experience clinical procedural skills during their early years of training. The findings of this study show that pre-clerkship procedural training facilitates the acquisition of clinical skills and improves students’ confidence. Most students found SBPT to be a useful and valuable learning method.

Though intriguing and unexpected, the findings we observed in the second phase of this study question the notion of skills retention and the value and adequacy of skill exposure to achieve procedural competency. Exploring this potential source of unique findings may hold answers to the questions our study brought forthwith, which may have significant implications and consequences on the current educational practices and educationists who seem reluctant to change.

### Supplementary Information

**Additional file 1.** 

## Data Availability

The datasets used and/or analyzed during the current study available from the corresponding author on reasonable request.

## Abbreviations

SBPT: Simulation-Based Procedural Training
IPPI: Integrated Procedural Performance Instrument
SP: Standardized patient
CAT: Communication Assessment Tool
OSCE: Objective Structured Clinical Examination
ALOBA: Agenda Led Outcome-Based Analysis scheme
MCQ: Multiple Choice Questions
DOPS: Direct Observation of Procedural Skills
OSATS: Observed Structured Assessment of Technical Skills
SS: Simulated setting
CS: Clinical setting
