# Supplementary material for: Pre-clerkship procedural training in venipuncture: a prospective cohort study on skills acquisition and durability
Source: BMC Med Educ. 2023 Oct 6;23:729. doi: 10.1186/s12909-023-04722-2 (PMC10559527; doi:10.1186/s12909-023-04722-2)
Supplement: Supplementary file 1 — Additional file 1. [file 12909_2023_4722_MOESM1_ESM.pdf]

Additional file 1

	BE*	B*	ME*	AE*	UC*
**Introduction/ establish rapport**					
Introduce yourself					
Patient identification					
Address concerns					
Reassure patient if anxious					
**Explanation of Procedure**					
Explain the procedure					
Check for understanding					
**Consent**					
Verbal consent					
**Preparation for procedure**					
Prepare all equipment					
Make patient comfortable sitting or lying down					
**Technical performance of the procedure**					
Assess patient’s antecubital fossa/forearm					
Select site for venepuncture					
Apply tourniquet					
Reassess vein and release tourniquet					
Hand hygiene, reapply tourniquet					

Disinfect entry site					
Insert the needle at 30-degree angle or less					
Draw blood (minimum 2 ml)					
Release tourniquet					
Remove needle					
Apply firm pressure on venepuncture site					
Cover site with dressing					
Fill the laboratory sample tubes					
**Maintenance of asepsis**					
Clean hands					
Put on gloves					
Disinfect venepuncture site					
Discard waste appropriately					
**Closure of procedure**					
Check venepuncture site for bleeding					
Ask for pain or discomfort					
Thank the patient					
Handover blood samples for transportation					
**Professionalism**					
**Overall ability to perform the procedure (Technical and professional skills)**					

*Response guide: BE- Below expectations, B- Borderline, ME- Meets expectations, AE- Above expectations, UC- Unable to comment
